# The Extract of* Fructus Psoraleae* Promotes Viability and Cartilaginous Formation of Rat Chondrocytes* In Vitro*

**DOI:** 10.1155/2016/2057631

**Published:** 2016-11-23

**Authors:** Xin Pan, Kang Xu, Xuefeng Qiu, Wen Zhao, Dong Wang, Li Yang

**Affiliations:** ^1^College of Pharmacy, South-Central University for Nationalities, Wuhan, China; ^2^National Innovation and Attracting Talents “111” Base, Key Laboratory of Biorheological Science and Technology, Ministry of Education, College of Bioengineering, Chongqing University, Chongqing, China; ^3^Department of Bioengineering, University of California at Los Angeles, 410 Westwood Plaza, 5121 Engineering V, Los Angeles, CA 90095, USA; ^4^Department of Cardiovascular Surgery, Union Hospital, Tongji Medical School, Huazhong University of Science and Technology, Wuhan, Hubei, China; ^5^School of Life Sciences, Northwestern Polytechnical University, Xi'an, Shaanxi, China

## Abstract

This study aimed to investigate the extract components of FP on rat chondrocyte function and cartilaginous formation* in vitro*. Petroleum ether extract (P-e) of FP extract components was selected to treat Sprague-Dawley rat chondrocytes. Cell viability was tested with different concentrations (0.1, 1, 10, and 100 *μ*g/mL) of P-e treatment. Concentrations of 0.1 and 1 *μ*g/mL P-e conditioned culture mediums were used for treating chondrocytes in experiments. Cell proliferation was measured via DNA incorporation assay. Type II collagen, aggrecan, and Sox-9 genes expression levels were measured with RT-PCR. Additionally, cartilaginous formation was analyzed with type II collagen immunofluorescence, H&E, and alcian blue staining. Concentrations of 0.1 and 1 *μ*g/mL P-e showed low cytotoxicity and demonstrated stimulatory effects on chondrocyte proliferation in early stages. Following 6 days of P-e culture, aggrecan and Sox-9 gene expression levels of the 1 *μ*g/mL P-e group were upregulated by 1.82- (*p* < 0.05) and 2.06-fold (*p* < 0.05), respectively, versus controls. Moreover, 1 *μ*g/mL P-e significantly stimulated cell aggregation and type II collagen deposits after 1 week of treatment. Noteworthy, tight cartilaginous structures formed in the 10-day 1 *μ*g/mL P-e conditioned culture. These findings suggest that P-e has the potential to treat cartilage degeneration induced by chondrocyte failure.

## 1. Introduction

Chondrocytes and cartilage matrix are important components of cartilage structure. Chondrocytes are highly specialized cells involved in synthesizing collagen and proteoglycans and the maintenance of cartilage homeostasis. Well-recognized cartilage diseases attributable to extracellular matrix (ECM) degeneration, which affect normal chondrocyte function, include osteoarthritis, achondroplasia, and articular cartilage injury [1, 2]. Importantly, chondrocyte death suppresses synthesis of the cartilage matrix resulting in delayed tissue remodeling and poor recovery function [3–5]. Further exacerbating these diseases affecting chondrocytes is the finding that chondrocyte proliferation is slow [6]. Therefore, effective pharmacologic therapy promoting chondrocyte proliferation and function may be an important target in treating cartilage disease.

In Traditional Chinese Medicine (TCM) theory,* Fructus Psoraleae* is used to treat a range of conditions including bone fractures and joint diseases. Phytochemical studies suggest that coumarins, flavonoids, and meroterpenoids are the primary active chemical components in FP [7, 8]. Several studies have observed that extract components of FP demonstrate a stimulatory effect on osteogenesis leading to new bone formation [9]. In particular, it has been reported that prenyl compounds relating to FP stimulate osteoblast proliferation and differentiation [10]. Additionally, our group previously demonstrated that psoralen, the main coumarins of FP, activates chondrocyte cartilaginous cellular functions [11]. Yang et al. also showed that psoralen regulates chondrocyte gene expression while also attenuating degeneration of intervertebral lumbar discs [12].

In this study, we identified and assessed extract components involved in stimulating chondrocyte proliferation and cartilaginous formation. After experiment screening, P-e of FP extract components was selected, and it showed low cytotoxicity and exhibited promotive effects on chondrocytes proliferation in the early stage. Moreover, P-e stimulated cell ECM gene expression, aggregation, depositing type II collagen, and forming a tight cartilaginous structure. These positive promotive effects on chondrocytes provide the basis for rechondrogenesis of injured cartilage. The P-e of FP could be further studied and developed some promising active medications for treating cartilage degeneration which is compromised by chondrocytes dysfunction.

## 2. Materials and Methods

### 2.1. Chondrocytes Isolation

Six healthy Sprague-Dawley rats were euthanized in order to identify and isolate chondrocytes for this study as described previously [11]. In brief, after cell isolation, chondrocytes were identified using type II collagen immunofluorescence (see Supplementary Figure 1 in Supplementary Material available online at http://dx.doi.org/10.1155/2016/2057631), while being cultured via culture medium consisting of low glucose Dulbecco modified Eagle medium (DMEM) supplemented with 10% FBS and 100 U/mL penicillin and 100 *μ*g/mL streptomycin. The third passage of cells was used for experiments.

### 2.2. Preparation and HPLC Analysis of P-e

*Fructus Psoraleae* was purchased from Sichuan Province Traditional Chinese Medicine Hospital and authenticated by Professor Xian-Ming Lu (Chengdu University of Traditional Chinese Medicine).* Fructus Psoraleae* was washed and decocted by refluxing twice with 75% ethyl alcohol (1 : 8, w/v), followed by water (1 : 10, w/v). Filtrates were combined and condensed to form a raw extract. Following this, raw extract was further extracted with petroleum ether using Soxhlet extraction and dried under reduced pressure to form the petroleum ether extract (yield: 5.28 ± 0.41%, *n* = 6, and percentage value represents extract/FP raw materials). The quality of FP extract was measured via HPLC. Briefly, 0.01 g P-e extract was dissolved in methanol and filtered using 0.45 *μ*m filters in preparation for HPLC analysis. Chromatographic conditions were Shimadzu LC-20AT-DAD HPLC system (Shimadzu Corp.), C18 column (4.6 × 250 mm, 5 *μ*m), injected volume of 10 *μ*L, column temperature of 30°C, flow velocity at 1 mL/min, UV wavelength at 230 nm, and gradient elution of acetonitrile/water (Supplementary Table 2).

### 2.3. Preparation of the Conditioned Culture Medium

P-e of FP was dissolved with DMSO to form stock solution (extracts concentration: 1 g/mL, expressed in the weight of raw materials per mL), sterilized using a 0.22 *μ*m aseptic filter, and stored at 4°C for later use. In preparing the conditioned culture medium, stock solution was added in low glucose DMEM supplemented with 10% FBS to form the conditional culture medium with the final concentration of 100 *μ*g/mL, 10 *μ*g/mL, 1 *μ*g/mL, and 0.1 *μ*g/mL (DMSO volume was 0.1% in the conditional culture medium). The blank contained untreated cells with low glucose DMEM supplemented with 10% FBS, whereas the control group contained the same proportion of DMSO compared to the test samples.

### 2.4. Cell Viability and Morphology Analysis

Cell viability was determined with the MTS assay. A standard curve was first established to test the linear relationship between cell number and optical density value. Following this, approximately 5 × 10^3^ chondrocytes were seeded in each well of a 96-well plate and then treated with a different conditional culture medium (P-e: 100, 10, 1, and 0.1 *μ*g/mL). After a culture period of 3 days, MTS colorimetric testing was performed each day to check OD values at 490 nm in each well. Additionally, cells were also microscopic observation and recording occurred to assess the effect of P-e on cell morphology on day 3.

### 2.5. EdU Incorporation Cell Proliferation Assay

Previous MTS viability assays showed cell viability and proliferation were optimal at the final concentration of 0.1 and 1 *μ*g/mL. Therefore, we also studied chondrocyte DNA synthesis using a treatment of 1 and 0.1 *μ*g/mL P-e extract for 24 hours via a Cell-Light EdU Apollo 488* in vitro* Imaging Kit. In brief, cells were seeded in 96-well plates at 5 × 10^3^ cells/well and then treated with 1 and 0.1 *μ*g/mL P-e extract conditioned culture medium for 24 hours. Following this culture period, cells were incubated with EdU-labeling medium (50 *μ*M) for 2 h. Thereafter, cells were detected using an Apollo fluorescent dye, counterstained with DAPI, and observed using a fluorescent microscope. The percentage of EdU-positive cells was calculated from five fields randomly selected in each well.

### 2.6. Cell Cycle Analysis

After 1 day of treatment at the final concentration of 0.1 and 1 *μ*g/mL, cells were trypsinized and then resuspended in PBS at 5 × 10^5^/mL, followed by fixation with 70% precooled ethanol overnight at 4°C. Fixed cells were centrifuged, washed, and stained with PI/RNase staining buffer for 1 h in a dark room at 4°C. Cell counts at different phases of cell cycle were analyzed by flow cytometry.

### 2.7. qRT-PCR Assay

Chondrocytes were seeded at a density of 1 × 10^5^ per well into 6-well plates. Cells were then cultured in normal culture medium or different conditioned medium. Gene expression was evaluated by qRT-PCR after 3 and 6 days. Total RNA was extracted using the total RNA extraction kit according to manufacturer's guidelines. First, strand cDNA synthesis was followed with the RT-PCR protocol. SD rat specific primers were designed using the NCBI Primer-BLAST and synthesized by Invitrogen (Table 2). The SsoAdvanced Universal SYBR Green Supermix and CFX96 Real-Time PCR Detection System were used to perform qRT-PCR. The real-time PCR condition was as follows: 95°C for 30 sec followed by 39 cycles of a two-temperature program for five sec at 95°C and 30 sec at 60°C. The gene expression levels were evaluated by the 2^−ΔΔct^ method.

### 2.8. Immunofluorescent Assay

Chondrocytes were seeded at a density of 1 × 10^4^ per well into 96-well plates. Cells were then cultured in normal culture medium or the final concentration of 0.1 and 1 *μ*g/mL conditioned medium for 7 days. Following this, cells were fixed with 4% PFA for 30 min, blocked with 1% bovine serum albumin for 3 min at room temperature, and stained with rabbit anti-type II collagen overnight at 4°C. After washing the cells with PBS, Alexa Fluor 488 dye-labeled goat anti-rabbit antibody was used as a secondary identifying antibody. Finally, cells were counterstained with DAPI.

### 2.9. Cartilaginous Formation Assay

Approximately 2 × 10^6^ chondrocytes were centrifuged in the bottom of each tube and then cultured at a final concentration of 1 *μ*g/mL conditioned culture medium and normal medium for 10 days to form the cartilaginous pellets. Thereafter, pellets were collected and fixed with liquid nitrogen, followed by frozen section processing. Section samples were stained with H&E and alcian blue prior to examination under the microscope.

## 3. Results

### 3.1. Identification of Main Components in FP P-e

**Figure 1:**
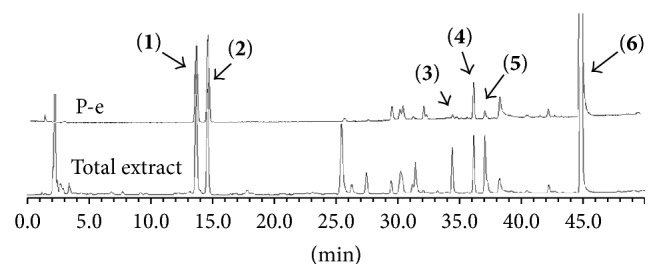
HPLC chromatogram for petroleum ether and total extract of* Fructus Psoraleae*. (**1**) Psoralen, (**2**) isopsoralen, (**3**) bavachin, (**4**) isobavachalcone, (**5**) bavachalcone, and (**6**) bakuchiol.

When compared with FP total extract, FP P-e contained less compounds. Six known main components in the FP P-e were found and identified by comparing the retention time with that of an accepted standard (Figure 1). The percentage content of (**1**) psoralen, (**2**) isopsoralen, (**3**) bavachin, (**4**) isobavachalcone, (**5**) bavachalcone, and (**6**) bakuchiol in the extract was 3.85%, 2.69%, 0.02%, 1.01%, 0.29%, and 84.5%, respectively (Table 1).

### 3.2. Effect of FP P-e Extract on Chondrocytes Morphology and Viability

**Figure 2:**
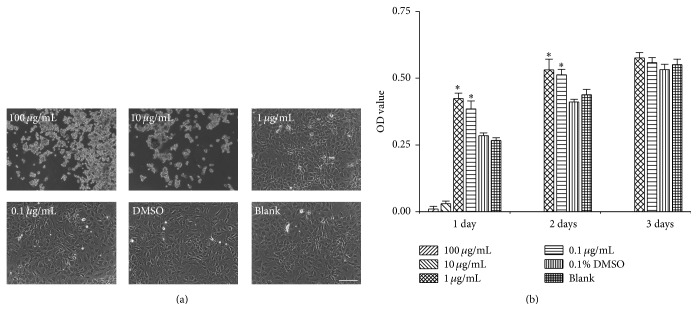
Effects of P-e treatments with different concentration on chondrocytes morphology and viability for 3 days. (a) Cell morphology under the phase contrast microscope. (b) Cell viability was tested via MTS assay, and data are presented as mean ± sd (*n* = 6), ^*∗*^ *p* < 0.05 versus DMSO group.

After a 3-day culture period, chondrocytes under 1 *μ*g/mL and 0.1 *μ*g/mL P-e conditioned culture mediums grew well and were polygonal, elliptical, and spindle-shaped (Figure 2(a)). However, the concentration of 100 *μ*g/mL and 10 *μ*g/mL showed increased cytotoxicity towards the chondrocytes leading to death (Figure 2: 100 *μ*g/mL and 10 *μ*g/mL). The OD value from MTS testing illustrates cell viability, while indirectly reflecting cell proliferation. Following a culture conditioning period of 3 days, P-e showed an increasing influence on cell proliferation at P-e concentrations of 1 *μ*g/mL and 0.1 *μ*g/mL (Figure 2(b)). In contrast, P-e concentrations of 100 *μ*g/mL and 10 *μ*g/mL led to profound cell death (Figure 2(b)). On day 1, 1 *μ*g/mL and 0.1 *μ*g/mL P-e significantly increased cell viability by 0.49- (*p* < 0.05) and 0.35-fold (*p* < 0.05) versus the control group (0.1% DMSO), respectively. On day 2, compared with the control group, cell viability of the 1 *μ*g/mL and 0.1 *μ*g/mL P-e extract increased by 0.29- and 0.25-fold, respectively. On day 3, the favorable effects of the P-e extract on cell viability plateaued. However, the OD value of 1 *μ*g/mL and 0.1 *μ*g/mL P-e extract treatments showed modest increases compared to the control group (*p* > 0.05).

### 3.3. Effect of FP P-e Extract on Chondrocytes Proliferation

**Figure 3:**
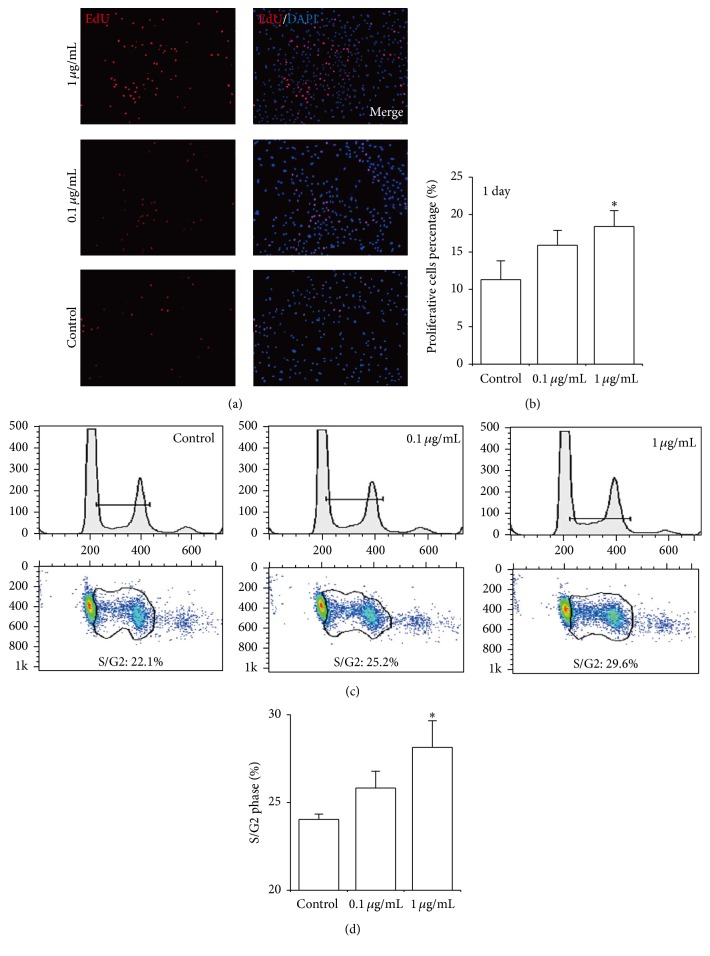
The effects of P-e on cell proliferation. (a and b) EdU incorporative DNA synthesis of rat chondrocytes with P-e treatment. (a) EdU staining (red) and DAPI (blue) in chondrocytes with P-e treatment for 24 h. (b) The histogram showed the percentage of EdU^+^ nuclei. Each bar represents the mean ± sd (*n* = 5); ^*∗*^ *p* < 0.05 versus DMSO group (control) was accepted as statistically significant. (c) Cell cycle by flow cytometry, indicating that 22.1%, 25.2%, and 29.6% of the cells were at the S/G2 phase for DMSO (control), 0.1 *μ*g/mL P-e, 1 *μ*g/mL P-e, respectively. (d) Statistical analyses for flow cytometry (*n* = 3); ^*∗*^ *p* < 0.05 versus DMSO group (control) was accepted as statistically significant.

The EdU incorporation assay illustrated cell DNA synthesis, showing the percentage of EdU^+^ nuclei was approximately 18.4%, 15.9%, and 11.3% for 1 *μ*g/mL, 0.1 *μ*g/mL, and control group, respectively, following 24 h culture periods (Figures 3(a) and 3(b)). Compared to the control group, the percentage of EdU^+^ nuclei at 1 *μ*g/mL and 0.1 *μ*g/mL P-e treatments increased by 0.63- and 0.41-fold, respectively (Figure 3(b)). Additionally, cell cycle detection showed that after 24 h culture periods with P-e, 29.6%, 25.2%, and 22.1% of cells were at the S/G2 phase for 1 *μ*g/mL, 0.1 *μ*g/mL concentrations, and control group, respectively (Figure 3(c)). The percentage of S/G2 cells at 1 *μ*g/mL P-e treatments showed significant difference versus control group (Figure 3(d)). Lastly, there was no proliferation stimulating effect of 3 days P-e treatment (Supplementary Figure 3). Thus, in the early cell cycle stages, P-e promoted cell proliferation via enhanced DNA synthesis and an increased cell cycle S/G2 phase.

### 3.4. Effect of FP P-e Extract on Chondrocytes Gene Expression and Cartilaginous Formation

**Figure 4:**
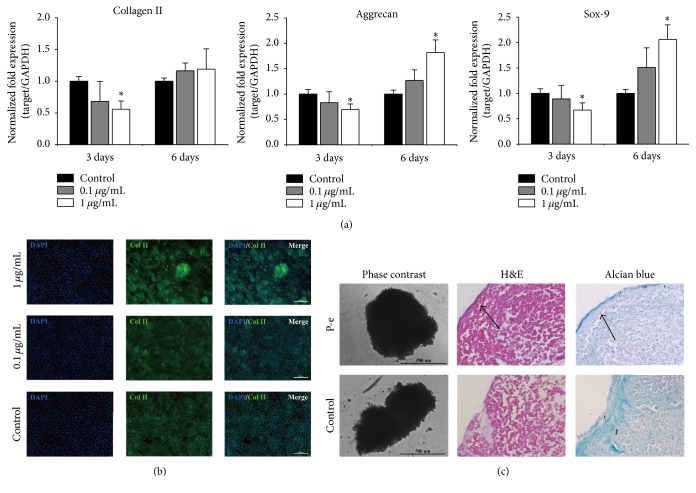
The effect of P-e on chondrocytes ECM gene expression and cartilaginous formation. (a) Gene expression level of different cartilaginous-related genes: collagen II, aggrecan, and Sox-9 at days 3 and 6 with P-e treatments. The data were analyzed by the 2^−ΔΔct^ relative quantification method (mean ± sd, *n* = 3). ^*∗*^ *p* < 0.05 versus DMSO group (control) was accepted as statistically significant. (b) Collagen II immunofluorescent staining of chondrocytes with P-e treatment for 1-week culture. (c) H&E and alcian blue staining of cartilaginous tissue sections of the pellet with P-e conditioned culturing for 10 days.

The gene transcript levels of collagen II, aggrecan, and SOX-9 of chondrocytes were analyzed via qRT-PCR for 3- and 6-day culture with normal culture medium and the conditioned culture medium with the final concentration of 0.1 *μ*g/mL and 1 *μ*g/mL P-e (Figure 4(a)). On day 3, compared with control group, the P-e concentration at 1 *μ*g/mL decreased type II collagen, aggrecan, and SOX-9 gene expression by 0.69- (*p* < 0.05), 0.56- (*p* < 0.05), and 0.67-fold (*p* < 0.05), respectively. Compared to the control, the P-e concentration at 0.1 *μ*g/mL decreased type II collagen, aggrecan, and SOX-9 gene expression by 0.11-, 0.17-, and 0.11-fold, respectively (all, *P* > 0.05). In contrast, on day 6, compared to the control group, aggrecan and SOX-9 gene expression in the 1 *μ*g/mL P-e group were upregulated by 1.82-fold (*p* < 0.05) and 2.06-fold (*p* < 0.05), respectively. Type II collagen gene expression demonstrated modest upregulation of 0.19-fold compared to controls (*p* > 0.05). Additionally, although not significant, 0.1 *μ*g/mL P-e leads to upregulation of type II collagen, aggrecan, and SOX-9 gene expression by 0.16-, 0.27-, and 0.51-fold, respectively.

Type II collagen immunofluorescent staining showed that 1 *μ*g/mL P-e significantly stimulated cell aggregation and type II collagen deposits (Figure 4(b)) compared to the control group after 1 week of conditioned culture.

Based on previous qRT-PCR and immunofluorescence analyses, 1 *μ*g/mL P-e was suggested to demonstrate the strongest effects on cartilaginous ECM gene expression. Cell pellets were cultured with 1 *μ*g/mL P-e conditioned culture medium for 10 days* in vitro*, and the cartilaginous constructions were evaluated by the frozen sections which were stained with H&E and alcian blue (Figure 4(c)). H&E staining showed that compared to the control group, the majority of chondrocytes aggregated towards the outer edge forming a cartilaginous rim in the P-e group (Figure 4(c): indicated by arrow); moreover the structure of the pellet of the P-e group was more dense and complete in shape (Figure 4(c)). Additionally, according to the alcian blue staining, stain density of the outer edge of the pellet was higher in the P-e extract group versus controls (Figure 4(c): indicated by arrow). This staining indicated that there were more glycosaminoglycan deposits in the P-e group. Lastly, we detected type II collagen with immunofluorescence on the frozen sections, while observing more type II collagen deposits in the P-e group compared to controls (Supplementary Figure 4).

## 4. Discussion

A lack of active blood supply within cartilage compromises the recovery of the cartilage degenerative diseases. Therefore, it is important to identify and develop targeted pharmacologic therapy that acts to inhibit cartilage failure while improving chondrocyte function. In previous studies from our group, we observed that psoralen promotes cartilaginous ECM synthesis, as well as increased cartilaginous gene expression. However, we found that psoralen does not simulate cell proliferation. In this study, we first in order prepared the solvent extracts of FP with petroleum ether, ethyl acetate, and n-butyl alcohol according to the chemical polarity (Supplementary Figure 2). Then, these different solvent extracts were screened with MTS assay as a pilot study to determinate the active extract for cell viability (data not shown). The findings of this pilot study showed that petroleum ether extract demonstrates a stimulating effect on cell viability, while exhibiting low cytotoxicity at concentrations of 0.1 and 1 *μ*g/mL. Thus, the petroleum ether extract was selected for ongoing experiments.

We acknowledge that extracts from herbal medicines, which are used in pharmacological studies, are limited by unstable quality control and undefined material basis of the chemical components. Therefore, in this study, we calculated the P-e extraction yield to confirm a stable and repeatable methodology for sample preparation. After HPLC analysis and identification, we identified the main components of P-e. We found 6 known compounds: psoralen, isopsoralen, bavachin, isobavachalcone, bavachalcone, and bakuchiol. Thus, quality control and HPLC analysis ensure a reliable P-e in future experiments.

It is worth noting that, in the extract, the percentage content of bakuchiol reached to 84.5%. Previous reports suggest that bakuchiol, which is a meroterpenoid, may play important roles in antimicrobial [13], antioxidant [14], antitumor [15], and inflammatory suppression activities [16]. Lim et al. compared different compounds from* Psoralea corylifolia* L. and demonstrated that bakuchiol is a key compound with estrogenic activity due to a high estrogen receptor-binding affinity [17]. Several studies have confirmed that estrogen and its similar active compounds regulate cell cycle for proliferation [18–21]. Therefore, we suggest that bakuchiol as an active compound demonstrating estrogenic activity may be used as a therapy for cartilage degenerative disease. Future studies are needed to investigate the potential role of bakuchiol as a therapy for cartilage degenerative disease.

Additionally, we observed that, with P-e treatment, cartilaginous gene (i.e., type II collagen, aggrecan, and Sox-9) expression levels were modestly downregulated in early phases, which was contrasted by upregulation in later phases. As such, we speculate that because cell proliferation is an energy intensive process that depends on cyclins and other regulated peptides, cells remaining in the S phase are not able to synthesize an extracellular matrix. Thus, in this study, expression levels of cartilaginous genes (i.e., type II collagen, aggrecan, and Sox-9 expression) were suppressed in early stages likely due to the effects of prior cell proliferation. Whereas another potential explanation is that some active compounds in P-e show demonstrate an inhibitory effect on ECM gene expression via high affinity receptors, others show stimulating effects due to low affinity receptors. Nevertheless, in supporting this hypothesis, future studies need to investigate different cell receptors signal transduction pathways involving each chemical compound associated with P-e.

In addition to the P-e stimulating effect on cell proliferation and gene expression, we also observed tight cartilaginous structures (i.e., pellets) in 1 *μ*g/mL P-e conditioned cultures that lasted 10 days. Thus, these data further support the suggestion that P-e treatment is able to promote cell proliferation as well as synthesize ECM for cartilaginous matrix formation. Although we did not study human chondrocytes, the present animal model observations provide a promising approach in developing new therapies for cartilage degenerative disease treatment. Future mechanism studies targeting human chondrocytes using the techniques in this study are warranted to improve the clinical translation of these data.

## 5. Conclusions

The results suggest that P-e may promote chondrocytes viability and proliferation, regulate cartilaginous extracellular matrix gene expression, and stimulate cartilaginous formation. These positive promotive effects on chondrocytes provide the basis for rechondrogenesis of injured cartilage. The P-e of FP could be further studied and developed some promising active medications for treating cartilage degeneration which is compromised by chondrocytes dysfunction.

## Supplementary Material


**Supplementary Figure 1**: Morphology and type II collagen immunofluorescence of rat chondrocytes.
**Supplementary Figure 2**: The solvent extracts of FP with petroleum ether, ethyl acetate, n-butyl alcohol and fraction according to the chemical polarity. The HPLC analysis of different fractions. (1) psoralen, (2) isopsoralen, (3) bavachin, (4) isobavachalcone, (5) bavachalcone, and (6) bakuchiol.
**Supplementary** **F** **igure 3**: Chondrocyte DNA synthesis with P-e treatment for 3 days. The histogram showed the percentage of EdU^+^ nuclei, each bar represents the mean ± sd (*n* = 5). There are no significant difference between groups.
**Supplementary** **F** **igure 4**: Type II collagen immunofluorescence on the frozen sections of pellets with P-e conditioned culture medium for 10 days *in vitro*. There are more type II collagen deposits in the P-e group compared to controls.
**Supplementary** **T** **able 1**: Yields of different fraction extract from .
**Supplementary** **T** **able 2**: Mobile phase elution procedure.

## Figures and Tables

**Table 1:** The molecular structure of compounds.

	Compound	Molecular structure	Content (%)
(**1**)	Psoralen	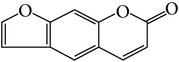	3.85%
(**2**)	Isopsoralen	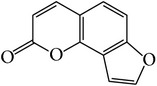	2.69%
(**3**)	Bavachin	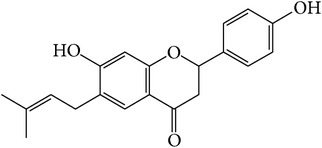	0.02%
(**4**)	Isobavachalcone	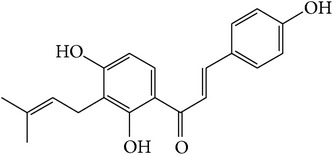	1.01%
(**5**)	Bavachalcone	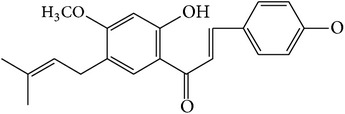	0.29%
(**6**)	Bakuchiol	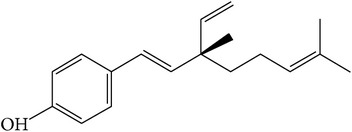	84.5%

**Table 2:** List of primer sequences for real-time PCR.

Gene name	Primers (5′-3′)	Production size (bp)
Collagen II	F: AATTTGGTGTGGACATAGGG	94
	R: AAGTATTTGGGTCCTTTGGG	
Aggrecan	F: GGTGGACCTGTAACAATCTT	509
	R: TCTTCTCCTGAGTATGAGGG	
SOX-9	F: AAGTGAAGGTAACGATTGCT	153
	R: CTCACTAACTCTGAAGGAGC	
GADPH	F: CAAGGTCATCCATGACAACT	253
	R: CAGATCCACAACGGATACAT	
