# Supplementary material for: The Extract of Fructus Psoraleae Promotes Viability and Cartilaginous Formation of Rat Chondrocytes In Vitro
Source: Evid Based Complement Alternat Med. 2016 Nov 23;2016:2057631. doi: 10.1155/2016/2057631 (PMC5141302; doi:10.1155/2016/2057631)
Supplement: Supplementary file 1 — Supplementary Figure 1: Morphology and type II collagen immunofluorescence of rat chondrocytes. Supplementary Figure 2: The solvent extracts of FP with petroleum ether, ethyl acetate, n-butyl alcohol and fraction according to the chemical polarity. The HPLC analysis of different fractions. (1) psoralen, (2) isopsoralen, (3) bavachin, (4) isobavachalcone, (5) bavachalcone, and (6) bakuchiol. Supplementary F igure 3: Chondrocyte DNA synthesis with P-e treatment for 3 days. The histogram showed the percentage of EdU+ nuclei, each bar represents the mean ± sd (n = 5). There are no significant difference between groups. Supplementary F igure 4: Type II collagen immunofluorescence on the frozen sections of pellets with P-e conditioned culture medium for 10 days in vitro. There are more type II collagen deposits in the P-e group compared to controls. Supplementary T able 1: Yields of different fraction extract from . Supplementary T able 2: Mobile phase elution procedure. [file 2057631.f1.docx]

**
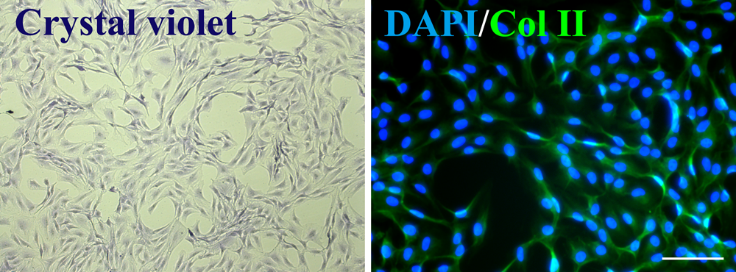
**

**Supplementary figure 1**. Morphology and type II collagen immunofluorescence of rat chondrocytes.

**
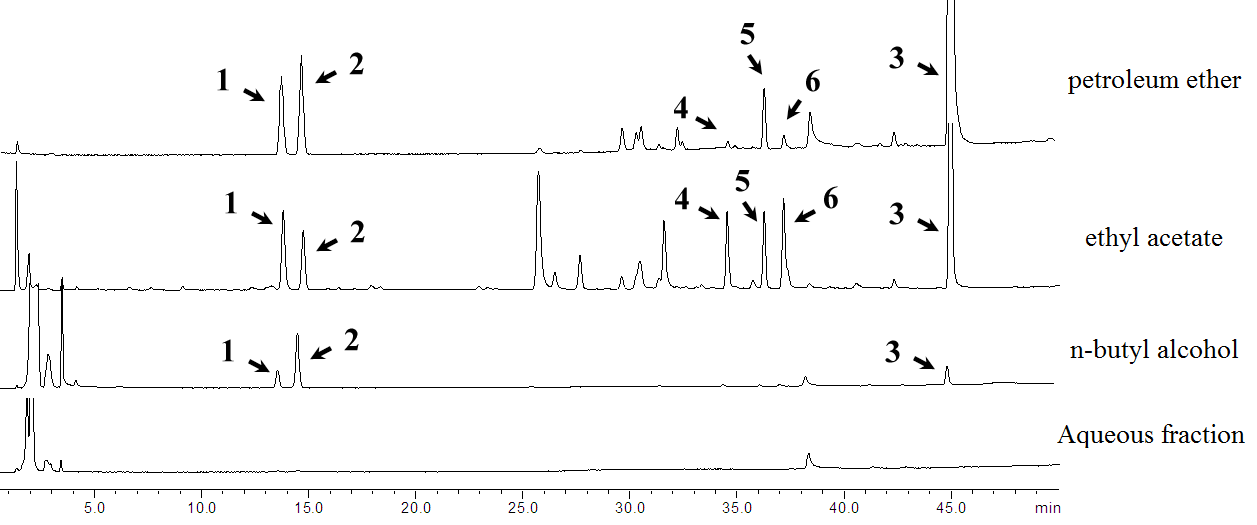
**

**Supplementary figure 2**. The solvent extracts of FP with petroleum ether, ethyl acetate, n-butyl alcohol and fraction according to the chemical polarity. The HPLC analysis of different fractions. (1) psoralen, (2) isopsoralen, (3) bavachin, (4) isobavachalcone, (5) bavachalcone, and (6) bakuchiol.

**
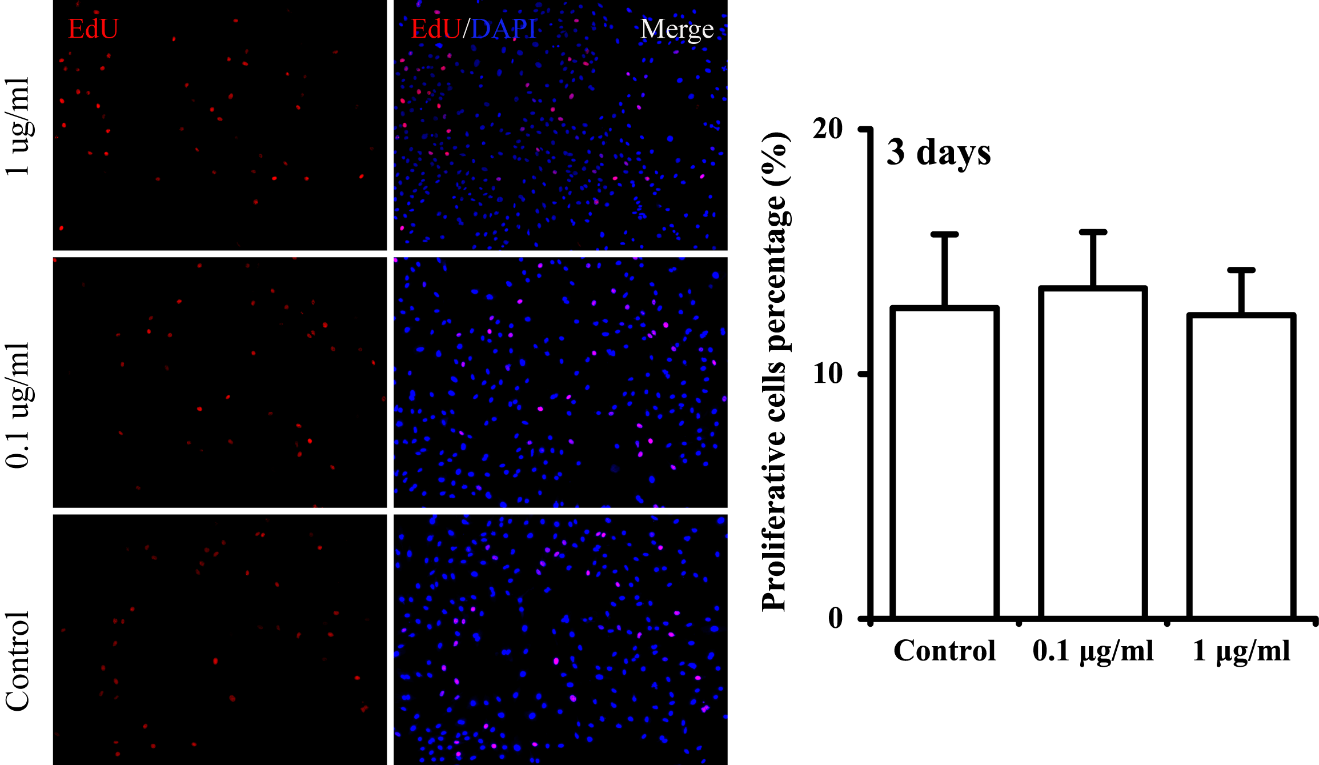
**

**Supplementary figure 3**. Chondrocyte DNA synthesis with P-e treatment for 3 days.

**
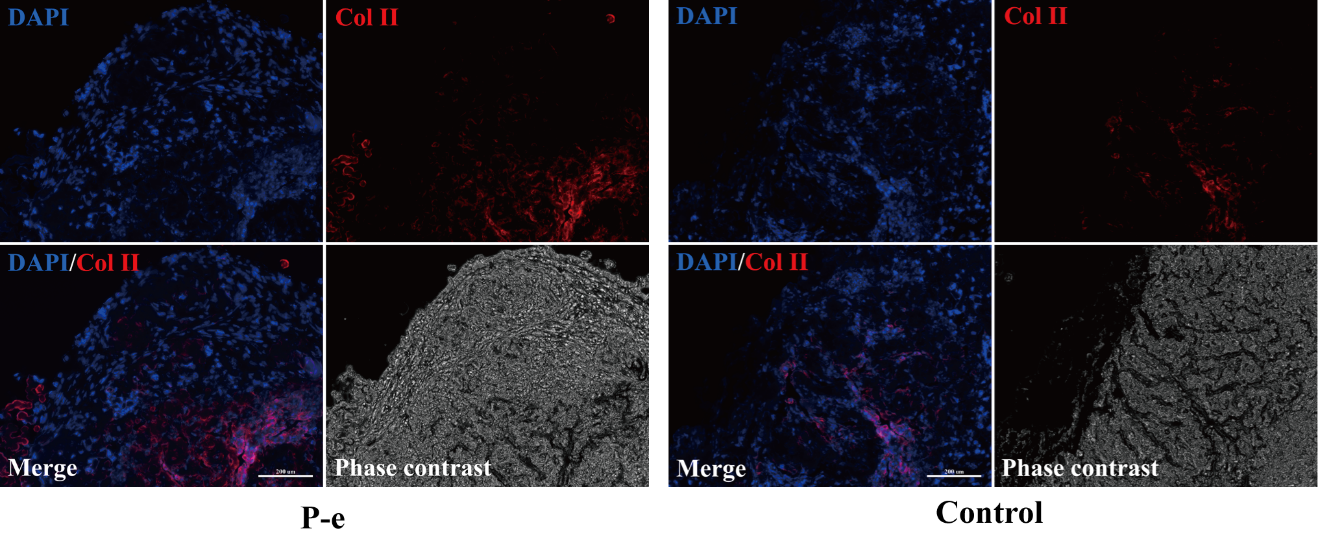
**

**Supplementary figure 4**. Type II collagen immunofluorescence on the frozen sections of pellets with P-e conditioned culture medium for 10 days *in vitro*.

Supplementary table 1. Yields of different fraction extract from [*fructus*](app:ds:fructus) [*psoraleae*](app:ds:psoraleae)

| Fractions | Extract yield (%) |
| --- | --- |
| Petroleum ether fraction | 5.28 ± 0.41 |
| Ethyl acetate fraction | 9.01 ± 0.46 |
| n-butyl alcohol fraction | 3.68 ± 0.48 |
| Aqueous fraction | 10.09 ± 0.59 |

Supplementary table 2. Mobile phase elution procedure

| time (min) | Acetonitrile (%) | water (%) |
| --- | --- | --- |
| 0 | 30 | 70 |
| 15 | 45 | 55 |
| 45 | 65 | 35 |
| 55 | 100 | 0 |
| 65 | 100 | 0 |
